# Estimated potential death and disability averted with vehicle safety interventions, Association of Southeast Asian Nations 

**DOI:** 10.2471/BLT.22.288895

**Published:** 2023-02-01

**Authors:** Jacobo Antona-Makoshi, Husam Muslim, Marko Medojevic, Sandra Watanabe, María Seguí-Gómez, Kavi Bhalla

**Affiliations:** a Japan Automobile Research Institute, 2530 Karima, Tsukuba, Ibaraki 305-0822, Japan.; b School of Engineering, Comillas Pontifical University, Madrid, Spain.; c Department of Public Health Sciences, University of Chicago, Chicago, United States of America.

## Abstract

**Objective:**

To evaluate road safety in member countries of the Association of Southeast Asian Nations and estimate the benefits that vehicle safety interventions would have in this group of countries.

**Methods:**

We used a counterfactual analysis to assess the reduction in traffic deaths and disability-adjusted life years (DALYs) lost if eight proven vehicle safety technologies and motorcycle helmets were entirely in use in countries of the Association of Southeast Asian Nations. We modelled each technology using country-level incidence estimations of traffic injuries, and the prevalence and effectiveness of the technology to calculate the reduction in deaths and DALYs if the technology was fitted in the entire vehicle fleet.

**Findings:**

The availability of electronic stability control, including the antilock braking systems, would provide the most benefits for all road users with estimates of 23.2% (sensitivity analysis range: 9.7–27.8) fewer deaths and 21.1% (9.5–28.1) fewer DALYs. Increased use of seatbelts was estimated to prevent 11.3% (8.11–4.9) of deaths and 10.3% (8.2–14.4) of DALYs. Appropriate and correct use of motorcycle helmets could result in 8.0% (3.3–12.9) fewer deaths and 8.9% (4.2–12.5) fewer DALYs.

**Conclusion:**

Our findings show the potential of improved vehicle safety design and personal protective devices (seatbelts and helmets) to reduce traffic deaths and disabilities in the Association of Southeast Asian Nations. These improvements can be achieved by vehicle design regulations and creating consumer demand for safer vehicles and motorcycle helmets through mechanisms such as new car assessment programmes and other initiatives.

## Résumé

**Objectif:**

Évaluer le niveau de sécurité routière des pays membres de l'Association des nations de l'Asie du Sud-Est et déterminer les avantages que procureraient des interventions de sécurité sur les véhicules pour ce groupe de pays.

**Méthodes:**

Nous avons procédé à une analyse contrefactuelle afin de mesurer la diminution du nombre de décès et d'années de vie ajustées sur l'incapacité (DALY) dus aux accidents de la route si huit technologies éprouvées en matière de sécurité des véhicules et casques pour motocyclistes étaient systématiquement d'application dans les pays appartenant à l'Association des nations de l'Asie du Sud-Est. Nous avons modélisé chaque technologie en nous fondant sur la fréquence estimée des blessures consécutives à des accidents de la route à l'échelle nationale, mais aussi sur la prévalence et l'efficacité de la technologie, dans le but de calculer la baisse des décès et DALY si l'ensemble de la flotte de véhicules était équipé de cette technologie.

**Résultats:**

C'est la disponibilité du contrôle électronique de la stabilité, systèmes de freinage antiblocage inclus, qui offrirait le plus d'avantages pour tous les usagers de la route avec, selon nos estimations, 23,2% (plage de l'analyse de sensibilité: 9,7–27,8) de décès en moins et 21,1% (9,5–28,1) de DALY en moins. Une utilisation accrue des ceintures de sécurité permettrait d'éviter 11,3% (8,11–4,9) de décès et 10,3% (8,2–14,4) de DALY. Enfin, l'emploi correct et approprié des casques pour motocyclistes pourrait entraîner une baisse de 8,0% (3,3–12,9) au niveau des décès et de 8,9% (4,2–12,5) au niveau des DALY.

**Conclusion:**

Nos résultats illustrent le potentiel que représente l'amélioration des systèmes de sécurité des véhicules et des dispositifs de protection individuelle (casques et ceintures de sécurité) dans la prévention des décès et handicaps liés aux accidents de la route au sein de l'Association des nations de l'Asie du Sud-Est. Ces améliorations peuvent être apportées en imposant une réglementation relative à la conception des véhicules ainsi qu'en encourageant les consommateurs à réclamer des véhicules et des casques plus sûrs pour motocyclistes, notamment par des mécanismes tels que des programmes d'évaluation des voitures neuves et d'autres initiatives.

## Resumen

**Objetivo:**

Evaluar la seguridad vial en los países miembros de la Asociación de Naciones de Asia Sudoriental y estimar los beneficios que tendrían las intervenciones en la seguridad de los vehículos en este grupo de países.

**Métodos:**

Se aplicó un análisis contrafáctico para evaluar la reducción de muertes por accidentes de tráfico y de años de vida ajustados por discapacidad (AVAD) perdidos si en los países de la Asociación de Naciones de Asia Sudoriental se emplearan en su totalidad ocho tecnologías de seguridad para vehículos y cascos de motocicleta que han demostrado su eficacia. Se modelizó cada tecnología utilizando estimaciones de incidencia de lesiones de tráfico a nivel nacional y la prevalencia y eficacia de la tecnología para calcular la reducción de muertes y AVAD si la tecnología se instalaba en todo el parque automovilístico.

**Resultados:**

La disponibilidad del control de estabilidad electrónico, incluidos los sistemas antibloqueo de frenos, proporcionaría los mayores beneficios para todos los usuarios de las vías públicas, con estimaciones de un 23,2 % (intervalo del análisis de sensibilidad: 9,7-27,8) menos de muertes y un 21,1 % (9,5-28,1) menos de AVAD. Se estimó que un mayor uso del cinturón de seguridad evitaría el 11,3 % (8,11-4,9) de las muertes y el 10,3 % (8,2-14,4) de los AVAD. El uso adecuado y correcto del casco de motociclista podría dar lugar a un 8,0 % (3,3-12,9) menos de muertes y un 8,9 % (4,2-12,5) menos de AVAD.

**Conclusión:**

Los resultados muestran el potencial de la mejora del diseño de la seguridad de los vehículos y de los dispositivos de protección personal (cinturones de seguridad y cascos) para reducir las muertes y discapacidades por accidentes de tráfico en la Asociación de Naciones de Asia Sudoriental. Estas mejoras pueden lograrse mediante normativas sobre el diseño de vehículos y la creación de una demanda de vehículos más seguros y cascos de motocicleta entre los consumidores a través de mecanismos como los programas de evaluación de vehículos nuevos y otras iniciativas.

## ملخص

**الغرض:**

تقييم سلامة الطرق في الدول الأعضاء في رابطة دول جنوب شرق آسيا، وتقدير الفوائد التي قد تعود من تدخلات سلامة المركبات على هذه المجموعة من الدول.

**الطريقة:**

قمنا باستخدام تحليل الواقع المضاد لتقييم الانخفاض في حالات الوفاة الناتجة عن حوادث المرور، ومعدلات سنوات العمر المعدلة بالإعاقة (DALY) المفقودة، في حالة إذا ما تم استخدام ثماني تقنيات مثبتة لسلامة المركبات وخوذات الدراجات النارية بالكامل في رابطة دول جنوب شرق آسيا. قمنا بوضع نموذج لكل تقنية باستخدام تقديرات الحدوث على مستوى الدولة للإصابات المرورية، وانتشار وفعالية التقنية لحساب الانخفاض في حالات الوفاة ومعدلات سنوات العمر المعدلة بالإعاقة (DALY)، إذا تم توظيف التقنية في أسطول المركبات بأكمله.

**النتائج:**

إن توفر التحكم في الثبات الإلكتروني، بما في ذلك أنظمة المكابح المانعة للانغلاق، يمكنه أن يوفر معظم الفوائد لجميع مستخدمي الطريق بتقديرات تصل إلى نسبة %23.2 (نطاق تحليل الحساسية: 9.7 إلى 27.8) أقل لحالات الوفاة، و%21.1 (9.5 إلى 28.1) أقل من معدلات سنوات العمر المعدلة بالإعاقة (DALY). تم تقدير زيادة في استخدام أحزمة الأمان لمنع %11.3 (8.11 إلى 4.9) من حالات الوفاة، و%10.3 (8.2 إلى 14.4) من معدلات سنوات العمر المعدلة بالإعاقة (DALY). يمكن أن يؤدي الاستخدام المناسب والصحيح لخوذات الدراجات النارية إلى تقليل حالات الوفاة بنسبة %8.0 (3.3 إلى 12.9)، وانخفاض معدلات سنوات العمر المعدلة بالإعاقة (DALY) بنسبة %8.9 (4.2 إلى 12.5).

**الاستنتاج:**

تُظهر النتائج التي توصلنا إليها إمكانيات التصميم المُحسّن لسلامة المركبات، وأجهزة الحماية الشخصية (أحزمة المقاعد والخوذات) لتقليل حالات الوفاة والإعاقات الناتجة عن الحوادث المرورية في رابطة دول جنوب شرق آسيا. يمكن تحقيق هذه التحسينات عن طريق لوائح تصميم المركبات، وخلق طلب من المستهلكين على المركبات الأكثر أمانًا، وخوذات الدراجات النارية، عن طريق آليات مثل برامج تقييم السيارات الجديدة، والمبادرات الأخرى.

## 摘要

**目的:**

评估东南亚国家联盟成员国的道路安全，并估计这些国家实施车辆安全干预措施后的益处。

**方法:**

我们使用了反事实分析法来评估在东南亚国家联盟的国家中完全使用八种经过验证的车辆安全技术和摩托车头盔的情况下交通事故死亡和伤残调整寿命年 (DALY) 损失的减少情况。我们使用国家级交通伤害发生率估计值和技术的普及率和有效性对每项技术进行建模，以计算在整个车队中纳入该技术后可减少的死亡和伤残调整寿命年情况。

**结果:**

电子稳定性控制（包括防抱死制动系统）的普及将为所有道路使用者带来最大的益处，估计能够减少 23.2%（敏感性分析范围：9.7-27.8）的死亡和 21.1% (9.5-28.1) 的伤残调整寿命年损失。据估计，增加安全带的使用可防止 11.3% (8.11-4.9) 的死亡和 10.3% (8.2-14.4) 的伤残调整寿命年损失。适当和正确佩戴摩托车头盔可减少 8.0% (3.3-12.9) 的死亡和 8.9% (4.2-12.5) 的伤残调整寿命年损失。

**结论:**

我们的研究结果表明，在东南亚国家联盟中，改进车辆安全设计和使用个人保护装置（安全带和头盔）有可能减少交通事故死亡和致残情况。这些改进可以通过制定车辆设计法规，和通过新车评估方案及其他举措等机制激发消费者对更安全车辆和摩托车头盔的需求来实现。

## Резюме

**Цель:**

Оценить безопасность дорожного движения в странах-членах Ассоциации стран Юго-Восточной Азии и определить возможные преимущества мероприятий по обеспечению безопасности транспортных средств в этой группе стран.

**Методы:**

Авторы использовали контрфактуальный анализ для оценки снижения смертности в результате ДТП и потерь лет жизни с поправкой на инвалидность (DALY), если бы в странах Ассоциации стран Юго-Восточной Азии полностью использовались восемь проверенных технологий безопасности транспортных средств и мотоциклетные шлемы. Каждая технология была смоделирована с использованием оценок случаев дорожно-транспортного травматизма на уровне страны, а также распространенности и эффективности технологии для расчета снижения смертности и DALY в случае внедрения технологии во всем автопарке.

**Результаты:**

Наличие системы курсовой устойчивости автомобиля, включая антиблокировочные тормозные системы, предоставит максимальные преимущества всем участникам дорожного движения: по оценкам, количество смертей сократится на 23,2% (диапазон анализа чувствительности: 9,7–27,8), количество DALY – на 21,1% (9,5–28,1). Более активное использование ремней безопасности, по оценкам, позволило избежать 11,3% смертей (8,11–4,9) и 10,3% DALY (8,2–14,4). Соответствующее и правильное использование мотоциклетных шлемов может привести к снижению смертности на 8,0% (3,3–12,9) и DALY на 8,9% (4,2–12,5).

**Вывод:**

Полученные результаты указывают на необходимость улучшения конструкции безопасности транспортных средств и средств индивидуальной защиты (ремней безопасности и шлемов) для снижения смертности и потери трудоспособности в результате дорожно-транспортных происшествий в странах Ассоциации стран Юго-Восточной Азии. Данные улучшения могут быть реализованы с помощью правил проектирования транспортных средств и создания потребительского спроса на более безопасные транспортные средства и мотошлемы с использованием таких механизмов, как программы оценки новых автомобилей и другие инициативы.

## Introduction

The Association of Southeast Asian Nations (ASEAN) is one of the world’s largest and fastest-growing economic regions. The association includes 10 countries with about 650 million people: Brunei Darussalam, Cambodia, Indonesia, Lao People's Democratic Republic, Malaysia, Myanmar, Philippines, Singapore, Thailand and Viet Nam. The economic development in the ASEAN has been accompanied by a rapid increase in motorized vehicles, with about 63 million passenger cars and 221 million motorcycles registered in 2019.^1^ This increase is reflected in the health burden caused by road traffic crashes in the ASEAN – 108 000 deaths in 2019.^2^

Unlike low- and middle-income countries, which account for 93% (1.25 million /1.35 million) of the world’s road deaths,^3^ high-income countries have succeeded in reducing traffic injuries.^4^ This reduction has been attributed to comprehensive efforts across a wide range of areas, including the strengthening of institutional capacity and road safety regulations, and improvements in medical care, road infrastructure and vehicle safety.^5^^,^^6^ Although member countries of the ASEAN have also implemented road safety interventions, these measures appear to have been insufficient.^7^ In 2020, United Nations (UN) Member States issued a General Assembly resolution requesting countries to halve road traffic deaths by 2030.^4^ Understanding what has been effective in high-income countries and what is unique to low- and middle-income countries is important to achieve this global target.

The effects of vehicle safety interventions on road traffic deaths and injuries are well established.^8^^,^^9^ Vehicle design improvements and vehicle safety interventions, such as antilock braking systems, electronic stability control, occupant restraints, airbags, side structure and padding, front-end design for pedestrian protection, and enforcement of the use of personal protective devices (for example, helmets and seatbelts), have proved to be effective in high-income countries. These measures have substantially reduced the risk of death and non-fatal injuries in vehicle occupants and vulnerable road users, such as pedestrians, bicyclists and motorcyclists.^10^^–^^21^

A few studies have estimated the effects of vehicle design improvements and the use of personal protective devices in low- and middle-income countries. They found that such measures reduced both deaths and injuries and that the cost would be small compared with the benefits.^22^^–^^25^ A recent study established that between 25% and 40% of all fatal road injuries worldwide could be averted by implementing four preventive interventions: speed restrictions, drink–driving ban, helmet use, and use of seatbelts and child restraints.^26^

Data on the effects of vehicle safety design and helmet use in the ASEAN are lacking. Therefore, we aimed to estimate the number of traffic deaths and disability-adjusted life years (DALYs) that could be averted if UN vehicle safety standards and the use of seatbelts and motorcycle helmets were widely implemented in the 10 member countries of the ASEAN.

## Methods

We used a counterfactual analysis^22^ to estimate the number of deaths, injuries and DALYs caused by road traffic incidents that would be averted if most of the vehicle and road users in member countries of the ASEAN adopted selected safety technologies and the use of personal protective devices. We estimated: (i) country-level incidence of traffic injuries; (ii) the prevalence of safety technologies and their use in each country; and (iii) changes in the incidence of traffic injuries if these technologies were fully implemented in member countries of the ASEAN based on relative risks obtained from literature reviews. We also assessed the influence of uncertainty on the estimates through a sensitivity analysis of the results based on alternative modelling assumptions.

### Deaths and injuries

In most low- and middle-income countries, official statistics on traffic injuries come from traffic police, which can result in substantial underreporting.^27^^–^^29^

**Fig. 1:**
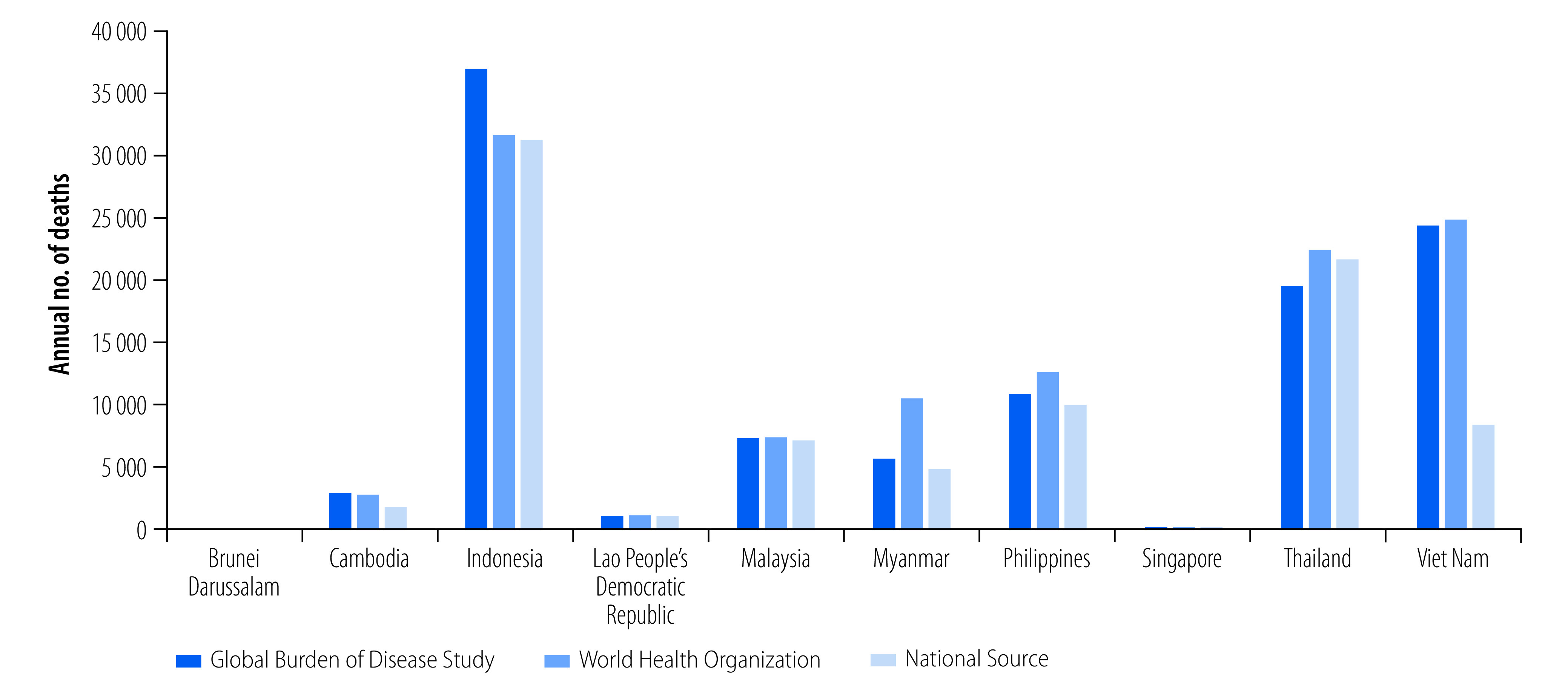
Total traffic deaths reported annually by country and source of data, 2018

Source: World Health Organization Global Status Report on Road Safety (2018) statistical annex^27^ and the Global Burden of Disease Study.^2^

Two global health statistical modelling studies provide estimates of road traffic deaths: the global health estimates of the World Health Organization (WHO)^3^ and the Global Burden of Disease Study.^2^ Both sources tend to give similar estimates of traffic deaths in member countries of the ASEAN (Fig. 1).

Although the Global Burden of Disease Study has reliable information on overall road traffic deaths and injuries, substantial discrepancies exist between the road user deaths reported in this study and the data from the official statistics of member countries of ASEAN. While the causes of the discrepancies are uncertain, previous studies have suggested poor coding quality for road-user types in death registration.^30^

To define the baseline traffic injury incidence data, we used the 2019 Global Burden of Disease estimates for total road traffic deaths and non-fatal injuries, disaggregated by age and sex, in each country.^2^ We further disaggregated the incidence data by road-user type (pedestrian, bicyclist, motorcyclist, occupant and other), using the proportions reported in each country (available in online repository).^31^ While the baseline death and injury data reported in the official statistics tend to be underreported, the reported proportions of deaths and injuries by road-user type are relatively accurate. Therefore, we used Global Burden of Disease data disaggregated by proportions of road-user type reported in the official statistics. In this way, we were able to overcome the problem of underreporting and provide an accurate proportion of deaths and injuries for each road-user type. However, official statistics for death proportions were only available for Malaysia, Singapore and Viet Nam. Therefore, we used 2016 death proportions reported in WHO’s 2018 report on road safety for the remaining countries.^27^ In the case of the Philippines, the WHO death proportions were considered unreliable, as 94% of road-user type deaths fell into the other category. For Brunei Darussalam and Lao People's Democratic Republic, death proportions were not available in either official statistics or the WHO report. Therefore, we used road-user type death proportions from the Global Burden of Disease Study for these three countries. For injury proportions, data were available only from the official statistics of Malaysia and Singapore. As the WHO report does not contain data on injury proportions, we used the proportions reported in the Global Burden of Disease Study.^2^

### Baseline burden

To estimate the current (baseline) burden in each country, we calculated DALYs based on the 2019 incidence data and proportions described in the previous section using a burden calculator tool.^32^ The calculator also requires population distribution data disaggregated by sex and age group. We identified national data sources for 2019 in Brunei Darussalam, Cambodia, Philippines and Singapore. We used World Bank data for the other countries.^33^

### Technology prevalence and use

We used a combination strategy of literature reviews, online searches and outreach to relevant institutions in the member countries of the ASEAN to obtain data on technology prevalence and use. We contacted more than 100 organizations and had technical discussions with experts from 24 institutions (online repository).^31^ The institutions were either relevant to a member country of the ASEAN (for example, Bureau of Philippine Standards and Malaysian Institute of Road Safety Research) or to the ASEAN as a whole (for example, New Car Assessment Program for Southeast Asian Countries).

We used country data for all technologies and safety interventions when available, and the average for other countries of similar income levels when not available (online repository).^31^

### Technology effectiveness

We used the results of a previous literature review to estimate the relative risk of death and injury associated with each technology.^22^ The original review excluded theoretical, modelling and laboratory studies, with a final selection of 13 papers from which estimates of technology effectiveness were extracted. We did a search using the same search strategy and inclusion criteria but found no new relevant publications. We also conducted a review of motorcycle helmet effectiveness (online repository).^31^ A summary of the sources of relative risk data is given in Box 1.

Box 1 **Source of risk used to estimate the effect of vehicle safety interventions on road traffic deaths and non-fatal injuries in member countries of the Association of Southeast Asian Nations**

***Antilock braking system***

An antilock braking system is a braking technology that prevents loss of steering control because of skidding in motorcycles and four-wheeled vehicles. The system uses sensors to detect locked wheels during braking manoeuvres and applies cycles of releasing, holding and reapplying brakes to allow the locked wheel to start rolling again.

*Vehicle occupants.* Relative risk (RR) of death and non-fatal injuries for occupants of cars and light trucks in run-off-road single-vehicle and multivehicle crashes reported by the National Highway Traffic Safety Administration, 2009;^34^ RR of deaths and non-fatal injuries for occupants of heavy vehicles in run-off-road single-vehicle and multivehicle crashes reported by National Highway Traffic Safety Administration, 2010.^35^

*Vulnerable road users.* RR of pedestrian death in vehicular crashes reported by the National Highway Traffic Safety Administration, 2009;^34^ RR of motorcyclist deaths;^14^ RR of motorcyclist non-fatal injuries from Spain, Italy and Sweden.^15^

***Electronic stability control***

Electronic stability control uses sensors to monitor the speed of each wheel to detect loss of traction and applies brakes to individual wheels. This feature helps the driver maintain control of the vehicle. All vehicles with electronic stability control are also equipped with an antilock braking system.

*Vehicle occupants*. RR of death and non-fatal injuries for occupants of cars, light trucks and heavy vehicles in the United States of America reported by the National Highway Traffic Safety Administration, 2015^36^.

*Vulnerable road users*. RR of death and non-fatal injuries for pedestrians based on data from the USA reported by the National Highway Traffic Safety Administration, 2015;^36^ RR of death and non-fatal injuries for motorcyclists as for antilock braking system.^14^^,^^15^

***Automobile seatbelts***

Seatbelts when properly fastened hold a person in place to avoid injuries in traffic incidents where an occupant could be thrown against a solid object.^37^The use of seatbelts reduces the user’s likelihood and severity of contact with the vehicle interior, distributes forces over wide parts of the body, and prevents the occupant being thrown from the vehicle.

*Vehicle occupants*. RR of death and injuries for occupants in frontal crashes.^38^

***Front airbags***

Front airbags complement seatbelts and prevent contact with the vehicle interior in frontal crashes.

*Vehicle occupants*. RR of death and non-fatal injuries for occupants in frontal crashes in the USA reported by the National Highway Traffic Safety Administration. 2015^36^ 

***Side airbags***

Side airbags prevent contact with vehicle interior in side crashes.

*Vehicle occupants*. RR of deaths of side crashes for head-and-torso airbags.^39^^,^^40^The same RR was applied to non-fatal injuries.

***Side door beams***

Side door beams provide additional structural integrity in side crashes.

*Vehicle occupants*. RR of deaths in side crashes in the USA reported by the National Highway Traffic Safety Administration, 2015.^36^ The same RR was applied to non-fatal injuries.

***Side structure and padding***

Side structure and padding reduce side impacts and increase energy absorption in side crashes.

*Vehicle occupants*. RR of deaths and non-fatal injuries in side crashes reported by the US National Highway Traffic Safety Administration, 2015.^36^

***Optimized system for side impact***

Optimized vehicle design ensures airbags work together with other design features in side crashes.

*Vehicle occupants*. RR of deaths in vehicles with the highest safety and performance rating, compared with vehicles rated the lowest.^12^

***Vehicle front-end design for pedestrian protection***

The designs modify the stiffness and energy absorption of the vehicle bumper, hood, windshield and A-pillar.

*Vulnerable road users*. RR of deaths^41^ and injuries^42^ for cars rated three or more stars versus zero stars in pedestrian protection tests of the European New Car Assessment Programme, and applied only to impacts with cars.

***Overall effects of vehicle design***

Estimates the combined effect of all the vehicle technologies.

*Vehicle occupants*. RR for occupant death between cars sold in the USA in 2015 versus in 1990 reported by the National Highway Traffic Safety Administration, 2015.^36^ The same RR was applied to non-fatal injuries.

*Vulnerable road users*. RR for pedestrian deaths^41^ and non-fatal injuries^42^ applied only to impacts with cars. RR for motorcycle deaths^12^ and non-fatal injuries^15^ for motorcycle antilock braking system.

***Motorcycle helmets***

Motorcycle helmets reduce the amount of energy transferred to the motorcyclist’s head in case of impact.

*Vulnerable road users*. RR of death and non-fatal injuries for motorcyclists.^43^^,^^44^

RR: relative risk.Notes: Vehicle occupant excludes motorcyclists. Vulnerable road users include pedestrians, cyclists and motorcyclists.

### Estimating deaths and DALYs

We used a comparative risk assessment approach to estimate the burden of injuries attributable to the lack of technology or unsafe practices, and the proportional reduction in mortality and morbidity if exposure to the risk factor was reduced in an alternative (counterfactual) scenario.^22^ The counterfactual scenario was defined as the availability of individual vehicle technologies in the entire fleet of vehicles. For seatbelts and helmets, the counterfactual scenario assumed that all traffic participants adhered to seatbelt and motorcycle helmet best practices. We modelled each intervention through the following steps: (i) identify which crash configurations are affected by the intervention; (ii) estimate relative risks in different crash configurations based on the literature review; (iii) estimate the proportion of traffic participants for whom the intervention applies; and (iv) estimate the number of lives saved, injuries prevented and DALYs averted if all vehicles were fitted with the standardized safety technologies, and motorized vehicle users adhered to the safety best practices.

For each risk factor related to safety technology, we defined the population attributable fraction (*PAF*) as the expected proportional reduction in mortality or morbidity if exposure to the risk factor were reduced to an alternative distribution, as shown in Equation 1.^22^ Thus, for a categorical variable, where *n* is the number of exposure categories (e.g. motorcycle helmet use), *P*_ί_ is the proportion of the population currently in the ίth exposure category (e.g. 80% of motorcyclists using a helmet), *Ṕ_ί_* is the proportion of the population in the i^th^ exposure category in the alternative scenario (e.g. 100% of motorcyclists using a helmet), and *RR_i_* is the relative risk of mortality or morbidity specific to the safety technology for each exposure category, for example, relative risk of death of motorcyclists involved in a crash with and without a helmet.
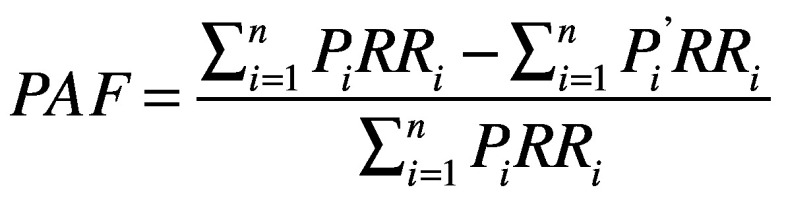
(1)Many safety technologies mitigate injuries in the same crash configuration and in combination with other technologies. For example, both seatbelts and front airbags reduce the risk of injuries in frontal traffic crashes, but their combined effect is interdependent. In other words, the cumulative effect of gains from individual technologies cannot be summed. Therefore, for non-motorized vulnerable road users (pedestrians and bicyclists), we applied the benefits of vehicle front-end design for pedestrian protection, excluding the benefits from other technologies, such as antilock braking systems and electronic stability control, because this pedestrian protection intervention is only available in passenger cars. Similarly, for motorized vulnerable road users (motorcyclists), we applied the benefits of motorcycle antilock braking systems and motorcycle helmets. For car occupants, we used estimates of the annual reduction in risk of occupant fatality in the United States of America as a result of vehicle design improvements, assuming that all motorized vehicles in member countries of the ASEAN had safety characteristics similar to those in the USA from 1980 to 2000.^36^

### Sensitivity analysis

In the sensitivity analysis, we recalculated the results based on alternative modelling assumptions. Box 2 summarizes input values for our main estimates and the sensitivity analysis. The values used for our calculations are available in the online repository.^31^ For baseline traffic injury estimates, we used maximum and minimum values of the 95% uncertainty interval of road injuries reported in the 2019 Global Burden of Disease Study,^2^ WHO data^3^ and national data estimates. For countries for which data on the prevalence of use of a particular technology was uncertain or not available, we used the maximum and minimum values based on neighbouring countries of similar income levels. We also estimated penetration of a particular technology based on the ratio of the presence of the technology in the top-selling cars divided by the total number of cars in the specified country. For technology effectiveness, we used the maximum and minimum values of the 95% confidence interval of the relative risk reported in previous studies selected from the systematic review. However, most of these effectiveness studies came from countries that adhered to road safety conventions and regulations. Thus, the effectiveness values may overestimate the actual effect of devices installed in vehicles in countries with no regulations in place. For example, to estimate the overall effect of vehicle design improvements, we followed the modelling of the baseline safety characteristics of the vehicle fleet based on the USA vehicle fleet in 1980 and 2000.^36^

Box 2 **Uncertainty of input data estimates and assumptions used to model the main estimates and the sensitivity analysis**

***Uncertainty in estimates of road traffic injuries at baseline***

Estimates of traffic deaths and injuries in member countries of the Association of Southeast Asian Nations vary substantially between sources.

- Main estimate modelling: overall road traffic injury incidence based on the Global Burden of Disease estimates from 2019.^2^
- Sensitivity analyses modelling: 95% uncertainty interval of Global Burden of Disease estimates from 2019,^2^ WHO’s global health estimates,^3^ and national estimates.
- Estimates of the distribution of incidence of injuries among road users (pedestrians, bicyclists, motorcyclists and vehicle occupants) vary substantially between Global Burden of Disease and national data estimates.
- Main estimate modelling: distribution of incidence of injuries from WHO’s global health observatory,^3^ and national data estimates.
- Sensitivity analyses modelling: 95% uncertainty interval of Global Burden of Disease estimates from 2019,^2^ WHO’s global health estimates,^3^ and national country estimates.

***Uncertainty in estimates of technology availability***

Information on the availability of technology in new vehicles sold was only available for Indonesia, Malaysia and Thailand for the year 2015.

- Main estimate modelling: estimated prevalence of technologies in vehicles in Indonesia, Malaysia and Thailand was based on a technology adoption model for each technology; for other countries, the average prevalence of these three countries was used.
- Sensitivity analyses modelling: for countries where the prevalence of technologies was unknown, minimum and maximum estimates were used by calculating the availability in new vehicle fleets between 2018 and 2019.

Seatbelt and motorcycle use estimates vary substantially between Global Burden of Disease^2^ and national estimates.

- Main estimate modelling: mean use estimate from the most robust evaluations for the main or best estimate.
- Sensitivity analyses modelling: minimum and maximum estimates of seatbelt and motorcycle helmet use from different data sources.
- Uncertainty in estimates of relative risk.

Significant variation exists in estimates of relative risk because of variation in quality and type of evaluations. The appropriate use of the intervention also causes variations.

- Main estimate modelling: mean relative risk estimate from the most robust evaluations for the main or best estimate.
- Sensitivity analyses modelling: minimum and maximum values of the 95% confidence interval of the relative risk (the specific modelling choices for each technology).

WHO: World Health Organization.

## Results

**Fig. 2:**
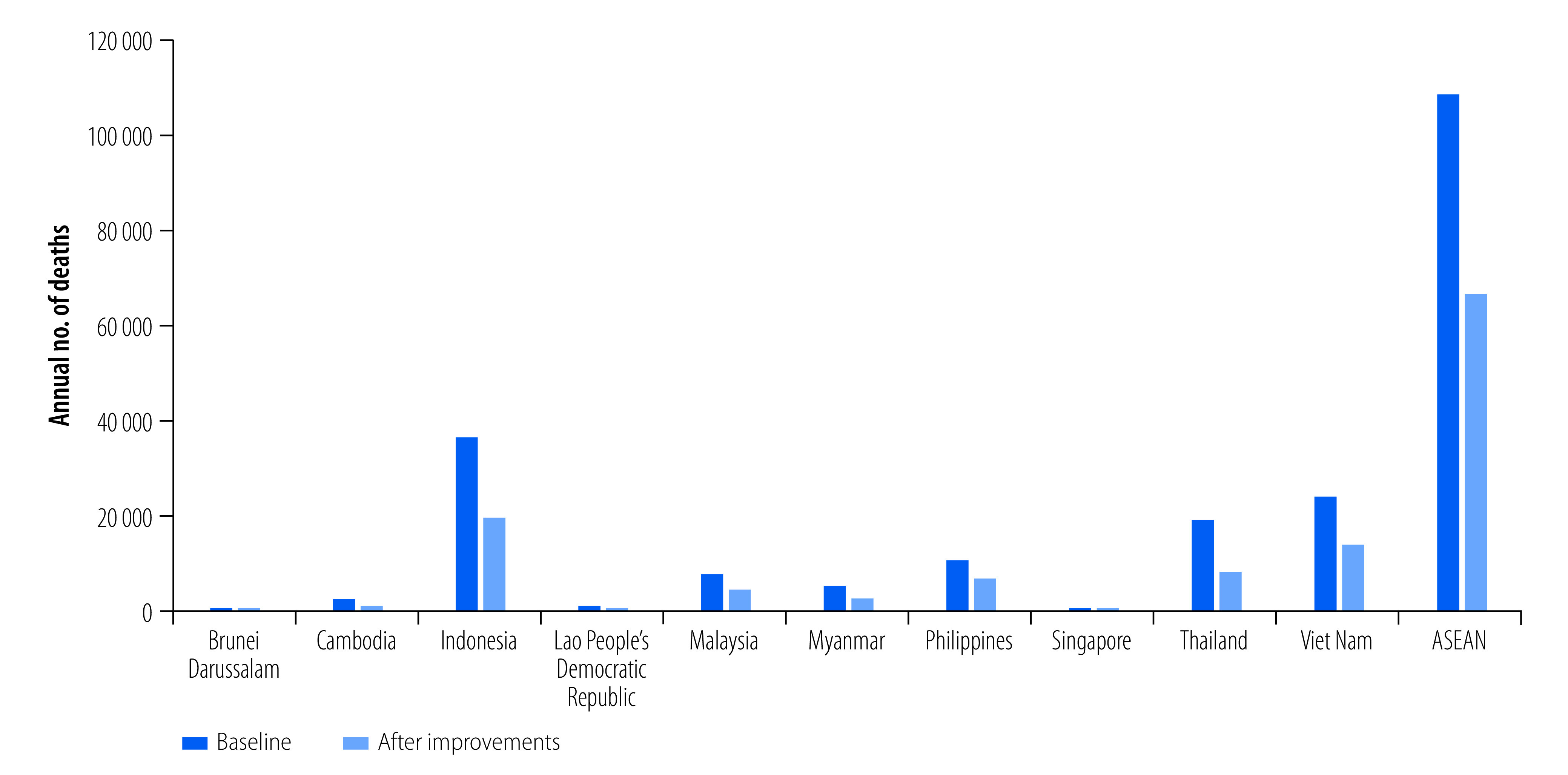
Estimated traffic deaths at baseline and with improved vehicle safety measures implemented and user adherence to safety best practices by country

**Fig. 3:**
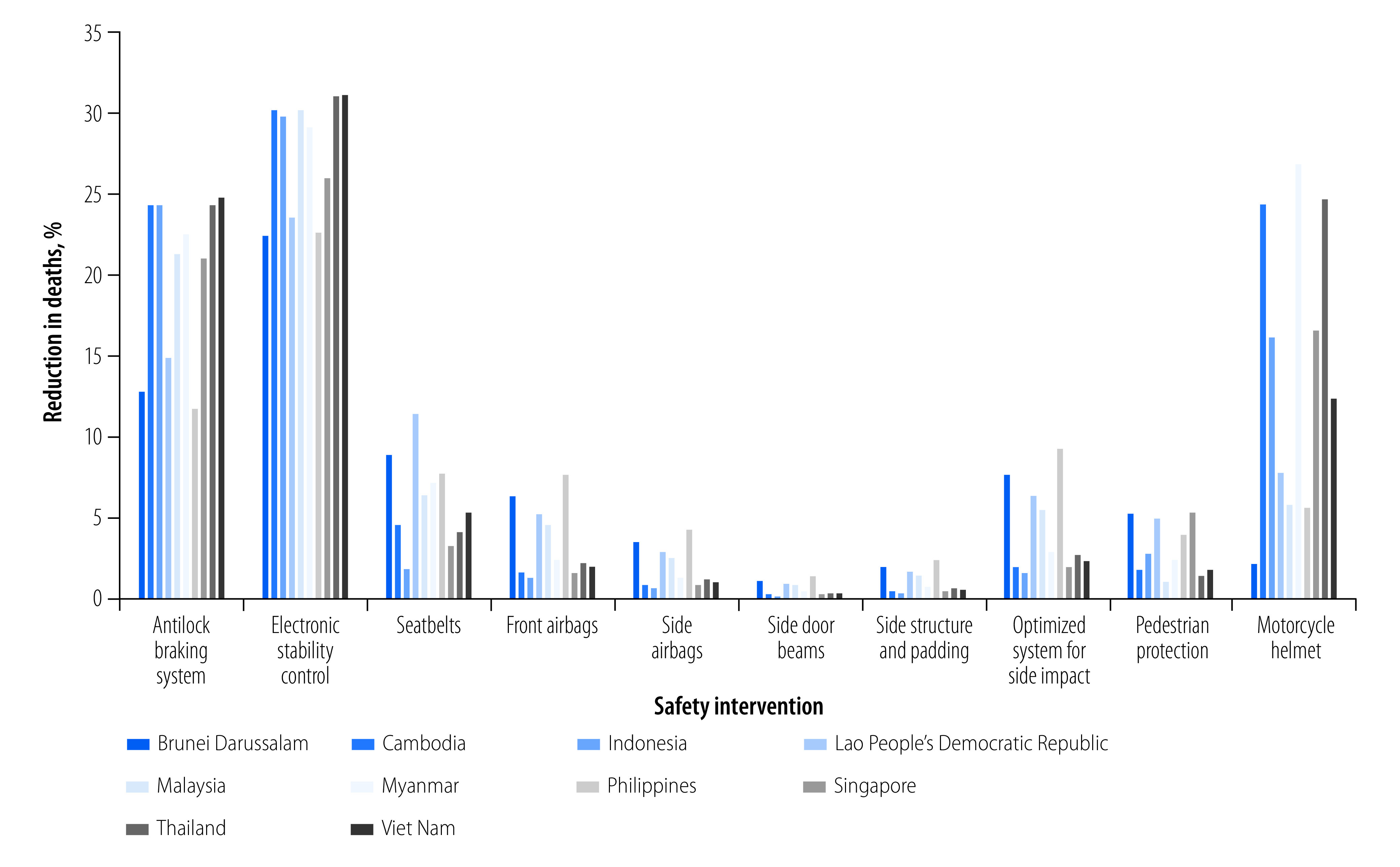
Estimated reduction in traffic deaths by road safety intervention and country

**Fig. 4:**
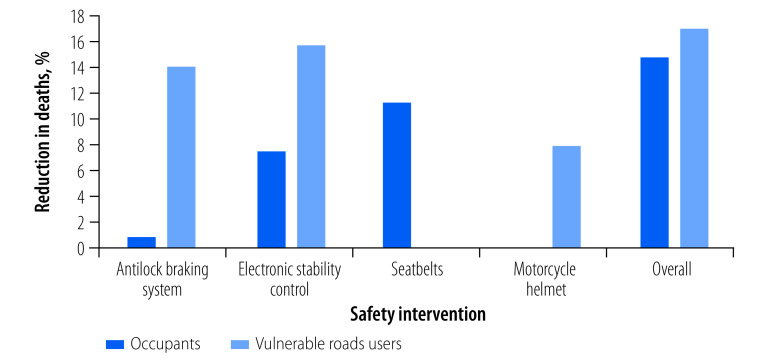
Estimated reduction in traffic deaths by vehicle safety intervention and type of road user

Note: Vulnerable road users include pedestrians, cyclists and motorcyclists.

A substantial number of lives would be saved by improved vehicle safety design and increased use of seatbelts and helmets in member countries of the ASEAN (Fig. 2). The estimated reductions in deaths and DALYs of each of the selected vehicle safety interventions are shown in Table 1 and Fig. 3, and Table 2, respectively. We disaggregated the reduction in deaths for vehicle occupants and vulnerable road users to show the safety benefits of the modelled interventions on different road users (Fig. 4).

**Table 1:** Deaths caused by traffic crashes and estimates of deaths averted through safety measures, by member country of the Association of Southeast Asian Nations

Parameter	Brunei Darussalam	Cambodia	Indonesia	Lao People's Democratic Republic	Malaysia	Myanmar	Philippines	Singapore	Thailand	Viet Nam	ASEAN
**Road user, no. of road traffic deaths, 2019**											
Pedestrian	18	289	5 859	299	455	799	2 416	48	1 561	2 413	14 158
Bicyclist	4	58	1 099	69	152	171	507	10	586	724	3 379
Motorcyclist	15	2 110	26 366	347	4 853	3 712	2 645	86	14 444	17 856	72 433
Occupant	24	289	2 930	346	2 123	857	5 069	16	2 733	2 896	17 281
Others	1	145	366	18	0	171	252	0	195	241	1 389
Total	61	2 891	36 619	1 080	7 582	5 710	10 889	159	19 518	24 130	108 639
**Safety intervention, estimated % of deaths averted (sensitivity analysis range)**											
Antilock braking system	12.8 (3.4–19.3)	24.3 (11.4–32.5)	24.2 (11.9–36.7)	14.8 (4.7–26.2)	21.3 (8.9–33.3)	22.5 (9.6–34.8)	11.8 (2.4–26.1)	21.0 (10.4–29.3)	24.3 (14.1–34.3)	24.8 (14.8–34.3)	14.9 (10.3–19.6)
Electronic stability control	22.4 (12.5–33.3)	30.2 (21.7–39.4)	29.8 (19.9–37.3)	23.5 (11.6–37.4)	30.1 (18.9–39.6)	29.1 (22.8–37.0)	22.6 (16.1–29.9)	26.0 (14.6–38.2)	31.0 (21.6–38.9)	31.1 (21.8–39.0)	23.2 (9.7–27.8)
Seatbelts	9.0 (6.1–11.0)	4.6 (2.4–6.9)	1.9 (1.2–2.6)	11.4 (8.8–13.9)	6.5 (4.2–8.3)	7.2 (4.9–10.2)	7.8 (3.9–8.9)	3.3 (2.6–4.2)	4.1 (2.4–5.1)	5.3 (3.2–6.8)	11.3 (8.1–14.9)
Front airbag	6.4 (4.2–7.3)	1.6 (0.8–2.0)	1.4 (0.8–1.8)	5.3 (2.4–6.7)	4.6 (3.1–6.3)	2.5 (2.0–2.9)	7.7 (4.2–8.8)	1.7 (0.3–2.1)	2.3(1.6–3.0)	2.0 (1.0–2.9)	4.3 (2.8–6.1)
Side airbags	3.6 (2.4–4.6)	0.9 (0.4–1.6)	0.7 (0.2–1.0)	3.0 (1.8–3.5)	2.6 (1.7–3.1)	1.4 (0.7–1.9)	4.4 (2.8–5.0)	0.9 (0.3–1.2)	1.3 (0.7–1.6)	1.1 (0.6–1.7)	3.1 (1.6–4.5)
Side door beams	1.2 (0.8–1.4)	0.3 (0.2–0.5)	0.3 (0.1–0.4)	1.0 (0.8–1.2)	0.9(0.5–0.9)	0.5 (0.2–0.5)	1.5 (1.0–1.8)	0.3 (0.1–0.4)	0.4 (0.3–0.5)	0.4 (0.1–0.5)	1.1 (0.8–2.0)
Side structure and padding	2.0 (1.8–2.4)	0.5 (0.3–0.6)	0.4 (0.3–0.5)	1.7 (0.9–1.9)	1.5 (1.0–1.6)	0.8 (0.4–0.8)	2.4 (1.9–2.7)	0.5 (0.2–0.6)	0.7 (0.5–0.8)	0.6 (0.3–0.8)	1.8 (1.1–2.7)
Optimized system for side impact	7.7 (3.7–8.2)	2.0 (1.0–2.1)	1.6 (0.7–1.9)	6.4 (3.2–7.3)	5.6(2.5–6.3)	3.0 (1.6–3.4)	9.2 (4.6–10.1)	2.0 (1.0–2.2)	2.8 (1.8–3.0)	2.4 (1.5–2.7)	6.7 (4.7–7.6)
Vehicle front-end design^a^	5.3 (4.3–9.5)	1.8 (0.8–3.2)	2.9 (2.0–5.1)	4.9 (3.1–9.7)	1.1 (0.7–2.9)	2.5 (1.8–5.2)	4.0 (2.2–6.7)	5.4 (4.0–10.3)	1.4 (0.8–2.9)	1.8 (1.0–3.1)	4.2 (3.0–5.2)
Motorcycle helmet	2.2 (0.8–8.6)	24.3 (12.3–33.8)	16.2 (8.9–28.4)	7.8 (2.1–19.5)	5.8 (1.4–15.7)	26.8 (13.6–37.7)	5.6 (2.0–14.5)	16.5 (8.1–24.9)	24.7 (12.1–36.3)	12.3 (3.9–27.5)	8.0 (3.3–12.9)
Overall^b^	30.6 (19.2–38.9)	29.1 (20.5–36.6)	29.2 (21.3–35.9)	29.9 (18.4–36.6)	33.4 (21.1–34.3)	29.7 (20.4–34.5)	32.5 (23.8–37.1)	27.5 (18.9–36.3)	31.0 (20.2–38.2)	30.5 (20.0–37.4)	30.7 (21.9–37.6)

^a^ For pedestrian protection.

^b^ Overall vehicle design improvement does not include the benefit of motorcycle helmets.

**Table 2:** DALYs lost because of traffic crashes and estimates of DALYs averted through safety measures, by member country of the Association of Southeast Asian Nations

Parameter	Brunei Darussalam	Cambodia	Indonesia	Lao People's Democratic Republic	Malaysia	Myanmar	Philippines	Singapore	Thailand	Viet Nam	ASEAN
**Road user, no. of DALYs lost, 2018**											
Pedestrian	815	16 234	348 578	15 684	23 296	45 354	130 711	1 967	81 705	124 396	1 275 614
Bicyclist	195	4 381	317 816	4 183	8 023	12 698	33 170	2 210	32 305	44 616	919 558
Motorcyclist	1 618	152 322	1 841 204	38 267	404 530	307 706	348 739	9 335	864 835	1 221 994	2 980 022
Occupant	1 579	32 581	361 635	27 026	151 777	93 502	356 323	3 323	216 183	300 256	2 378 474
Other	37	6 597	53 479	856	0	7 915	12 178	113	8 588	9 477	172 299
Total	4 245	212 115	2 922 711	86 016	587 625	467 175	881 121	16 947	1 203 616	1 700 738	7 725 967
**Safety intervention, estimated % of DALYs averted (sensitivity analysis range)**											
Antilock braking system	14.4 (5.6–21.5)	22.8 (11.6–33.8)	19.7 (10.3–30.0)	16.1 (7.1–23.6)	21.7 (11.4–30.7)	21.2 (10.5–31.2)	14.1 (6.5–23.3)	16.7 (8.8–25.9)	22.9 (12.3–32.7)	22.7 (11.2–32.0)	13.4 (10.2–18.8)
Electronic stability control	24.0% (8.8–33.5)	29.4 (10.0–37.2)	25.1 (11.3–34.5)	24.8 (9.4–36.4)	29.9 (12.7–36.5)	28.4 (11.7–36.9)	24.1 (10.1–34.6)	22.9 (12.3–32.9)	29.9 (14.0–36.8)	29.8 (14.7–36.1)	21.1 (9.5–28.1)
Seatbelts	8.6 (5.7–10.4)	7.0 (4.3–9.3)	3.0 (2.1–3.7)	11.1 (9.1–14.0)	6.0 (4.0–7.8)	9.6 (6.3–12.9)	6.7 (3.6–9.0)	6.5 (2.9–8.5)	5.3 (2.3–7.4)	7.8 (3.6–9.4)	10.3 (8.2–14.4)
Front airbag	5.1 (2.7–8.4)	1.8 (0.9–2.5)	1.4 (0.7–2.1)	4.2 (3.0–5.3)	3.4 (2.1–5.7)	2.3 (1.1–5.0)	5.6 (8.4–3.4)	1.9 (1.0–2.9)	2.3 (1.4–4.8)	2.0 (0.7–3.5)	3.3 (2.6–6.6)
Side airbags	3.8 (2.5–3.9)	1.7 (0.9–1.8)	1.4 (0.8–1.9)	3.3 (2.2–3.8)	2.7 (1.9–3.1)	2.2 (1.7–2.7)	4.1 (3.0–4.9)	2.3 (1.9–3.0)	1.9 (1.0–2.7)	2.0 (1.0–3.0)	3.1 (1.1–4.7)
Side door beams	1.3 (0.7–1.8)	0.6 (0.1–0.9)	0.5 (0.0–0.8)	1.1 (0.3–1.5)	0.9 (0.3–1.5)	0.7 (0.2–1.0)	1.4 (0.7–2.0)	0.8 (0.2–0.9)	0.6 (0.2–0.9)	0.7 (0.2–0.8)	1.1 (0.6–2.2)
Side structure and padding	2.1 (1.3–2.8)	1.0 (0.3–1.7)	0.8 (0.2–1.0)	1.9 (0.9–2.8)	1.5 (0.6–2.2)	1.3 (0.6–2.1)	2.3 (1.3–2.9)	1.3 (0.5–2.0)	1.0 (0.4–1.5)	1.1 (0.7–1.6)	1.8 (0.8–2.9)
Optimized system for side impact	8.1 (3.2–9.0)	3.7 (1.4–4.1)	3.0 (1.2–3.7)	7.0 (2.9–7.9)	5.8 (2.1–6.8)	4.7 (2.1–5.3)	8.9 (3.8–9.6)	5.0 (2.3–6.9)	4.1 (1.9–6.2)	4.2 (1.9–6.0)	6.7 (4.1–7.5)
Vehicle front-end design^a^	3.4 (1.8–4.9)	1.4 (0.6–2.2)	2.1 (1.4–2.8)	3.3 (1.6–4.5)	0.7 (0.2–1.6)	1.7 (1.0–2.3)	2.6 (1.8–3.3)	2.1 (0.9–3.1)	1.2 (0.5–2.1)	1.3 (0.4–1.9)	2.9 (2.8–5.9)
Motorcycle helmet	3.5 (1.1–8.2)	24.3 (17.4–32.7)	14.2 (8.9–22.4)	10.8 (6.7–20.0)	6.3 (2.3–13.0)	27.1 (18.4–34.9)	9.1 (4.4–15.7)	16.8 (9.4–24.6)	24.0 (12.8–31.4)	12.0 (5.7–16.8)	8.9 (4.2–12.5)
Overall^b^	31.7 (19.6–37.8)	30.3 (21.2–37.2)	28.7 (21.6–35.6)	31.1 (19.1–35.4)	33.0 (21.0–34.7)	30.9 (21.3–35.7)	32.6 (24.2–37.9)	29.3 (19.6–36.4)	31.4 (21.4–37.3)	31.3 (20.1–37.1)	30.1 (22.1–36.8)

DALYs: disability adjusted life years.

^a^ For pedestrian protection.

^b^ Overall vehicle design improvement does not include the benefit of motorcycle helmets.

The improvements in vehicle safety design and personal protective devices may save more than 42 000 lives (Fig. 2) and reduce the burden of traffic incidents by more than 3 013 127 DALYs (Table 2) in the ASEAN every year. The percentage reduction is greater in countries with more deaths (online repository).^31^ The increased use of antilock braking systems would result in 14.9% (sensitivity analysis range: 10.3–19.6; Table 1) fewer road traffic deaths and 13.4% (sensitivity analysis range: 10.2–18.8; Table 2) fewer DALYs. The gains would be comparatively small for vehicle occupants and substantial for vulnerable road users (Fig. 4): 13.9% fewer deaths in vulnerable road users compared with < 2% fewer deaths in vehicle occupants. These benefits were more significant in countries with a higher incidence of motorcyclist and pedestrian injuries (Cambodia, Indonesia, Malaysia, Myanmar, Singapore, Thailand and Viet Nam) than in countries with fewer motorcyclist and pedestrian injuries (Brunei Darussalam, Lao People's Democratic Republic and Philippines; online repository).^31^ The fitting of electronic stability control, which incorporates antilock braking systems, in all vehicles would provide the greatest benefits for all road users, estimated at 23.2% (sensitivity analysis range: 9.7–27.8) fewer deaths and 21.1% (sensitivity analysis range: 9.5–28.1) fewer DALYs.

Increasing seatbelt use to 100% would reduce deaths by 11.3% (sensitivity analysis range: 8.1–14.9) and DALYs by 10.3% (sensitivity analysis range: 8.2–14.4), but these estimates vary substantially between countries. For example, in Indonesia, which had a small proportion of occupant injuries (8%) and a comparatively high seatbelt use (69%), overall deaths and DALYs would be reduced by 1.9% (sensitivity analysis range: 1.2–2.6) and 3.0% (sensitivity analysis range: 2.1–3.7), respectively (online repository).^31^ In contrast, in Lao People's Democratic Republic, which had a relatively high proportion of vehicle occupant injuries (30%) and a low seatbelt use (45%), overall deaths and DALYs would be reduced by 11.4% (sensitivity analysis range: 8.8–13.9) and 11.1% (sensitivity analysis range: 9.1–14.0), respectively (online repository).^31^ Similarly, increasing front airbags would result in 4.3% (sensitivity analysis range: 2.8–6.1) fewer deaths and 3.3% (sensitivity analysis range: 2.6–6.6) fewer DALYs in the ASEAN.

Of the three side-impact technologies assessed (side airbags, side door beams and side structure with padding), side airbags would result in the greatest reduction in deaths, 3.1% (sensitivity analysis range: 1.6–4.5). These reductions varied depending on the proportion of vehicle occupant injuries in each country. Side structure with padding, which reduced deaths by 1.8% (sensitivity analysis range: 1.1–2.7) and DALYs by 1.8% (sensitivity analysis range: 0.8–2.9), was more beneficial than side door beams. However, integrating these technologies into a system that optimizes their overall benefits would result in greater reductions than each technology alone, with 6.7% (sensitivity analysis range: 4.7–7.6) fewer deaths and 6.7% (sensitivity analysis range: 4.1–7.5) fewer DALYs.

Improving vehicle front-end design for pedestrian protection would result in 4.2% (sensitivity analysis range: 3.0–5.2) fewer deaths and 2.9 (sensitivity analysis range: 2.8–5.9) fewer DALYs in the ASEAN. The benefits would be most noticeable in countries where pedestrian injuries are greater than for other road users. For instance, in Brunei Darussalam, deaths and DALYs would be reduced by 5.3% (sensitivity analysis range: 4.3–5.9) and 3.4% (sensitivity analysis range: 1.8–4.9), respectively. However, the gains in Cambodia, Malaysia, Thailand and Viet Nam, where fewer traffic injuries involve pedestrians, would be less than 2%.

Increasing motorcycle helmet use would reduce total deaths by 8.0% (sensitivity analysis range: 3.3–12.9) and DALYs by 8.9% (sensitivity analysis range: 4.2–12.5) in member countries of the ASEAN with significant variability between countries. In Brunei Darussalam, which has a low proportion of motorcyclist deaths (24%) and high helmet use (90%; online repository),^31^ the overall deaths and DALYs would decrease by 2.2% (sensitivity analysis range: 0.8–8.6) and 3.5% (sensitivity analysis range: 1.1–8.2), respectively. In contrast, in Thailand, which has a large proportion of motorcyclist fatalities (74%) and a relatively low helmet use (50%; online repository),^31^ the overall deaths would fall by 24.7% (sensitivity analysis range: 12.1–36.3) and DALYs by 24.0% (sensitivity analysis range: 12.8–31.4).

## Discussion

We aimed to estimate traffic deaths and DALYs that could be averted if the priority UN vehicle safety standards were widely implemented in the 10 member countries of the ASEAN. All the safety interventions assessed have been available for many years and the reductions in traffic deaths and injuries they bring about have been well established in robust epidemiological evaluations. Our results show that wider availability and use of these interventions would substantially reduce traffic deaths and injuries in member countries of the ASEAN. However, our findings might underestimate the real effects if the whole vehicle fleet in member countries of the ASEAN were to meet recognized international standards for vehicle safety because availability of some of these technologies could be low.

The benefits of vehicle safety interventions would be greater for vulnerable road users than for vehicle occupants. The antilock braking systems and electronic stability control technologies, proven essential for vehicle stability during braking manoeuvres and negotiation of bends, can save significantly more vulnerable road users (pedestrians, bicyclists and motorcyclists) than vehicle occupants. There are two likely reasons for this finding. First, the proportion of vehicle occupants in member countries of the ASEAN is significantly less than the proportion of vulnerable road users. Second, the risk of sustaining injuries in a crash is much lower for vehicle occupants than vulnerable road users.

An increase in vehicle production costs is commonly used to justify not implementing safety technologies in low- and middle-income countries. Although little public evidence is available on how incorporation of these regulations and technologies would affect vehicle prices and consumer decisions, estimates suggest that the cost of these technologies is about 50 United States dollars (US$) for antilock braking systems and US$ 75–100 for electronic stability control.^24^ Furthermore, global initiatives have shown that it is possible to produce and certify low-cost helmets that pass the major standards tests for less than US$ 20.^45^ The public health benefit associated with the wide implementation of these technologies overrides their implementation cost.^24^

Our results on the benefits of motorcycle helmets may underestimate the actual potential benefits of wider and correct helmet use. The effectiveness of helmets in protecting against injury is affected by their quality and correct use.^46^ Furthermore, uncertified or substandard helmets are commonly reported in member countries of the ASEAN. Therefore, contrary to expectations, our results suggest that seatbelt use would bring more benefits to occupants of four-wheeled vehicles than helmet use brings to motorcyclists. Although the relative risk of injury associated with seatbelt use is higher than motorcycle helmets (that is, the protection provided by helmets to motorcyclists is higher than that provided by the seatbelts to vehicle occupants), the baseline seatbelt use rates are lower than baseline helmet use rates. Our study included helmet-use data from reliable and nationally representative sources, but these data did not allow us to differentiate between motorcyclists with incorrect helmet usage or substandard helmets and those with correct helmet usage or certified helmets. Hence, many motorcyclists considered to be correctly wearing helmets in our models might have been misclassified, resulting in an overestimate of the helmet use in our main model and an underestimate of their benefits. Studies that consider correct helmet use and helmet quality would help to refine the estimations of the benefits of wearing helmets.

A limitation of this study is the lack of sources of information on the availability of the safety technology in several countries, which may decrease the benefits estimated for those countries. However, our analyses used numerous data from many sources. The most difficult data to obtain were on the safety equipment of vehicles in the countries. Government vehicle registries did not have this information and manufacturers rarely provided it. The gap in this information needs to be filled, given the public health burden caused by the use of motorized vehicles. We recommend that manufacturers and governments produce these data more readily. Standardized data will allow governmental or nongovernmental organizations to create a public database that identifies which car models have safety technologies, which will help vehicle users make their decision based on safety and cost.

To reach the UN target to halve traffic deaths by 2030 in the member countries of the ASEAN, plans are needed to ensure vehicle safety and the use of personal protective devices. Unless governments and industries rapidly enforce vehicle safety interventions, this target will not be attained. Most of the interventions included in our study have been available for more than 50 years, and international vehicle regulations have been in place since 1958. The time to act thus seems long overdue; hence it is imperative to take action now.
